# Vaccination reduces need for emergency care in breakthrough COVID-19 infections: A multicenter cohort study

**DOI:** 10.1016/j.lana.2021.100065

**Published:** 2021-09-09

**Authors:** Amit Bahl, Steven Johnson, Gabriel Maine, Martha Hernandez Garcia, Srinivasa Nimmagadda, Lihua Qu, Nai-Wei Chen

**Affiliations:** 1 Department of Emergency Medicine, Beaumont Hospital, Royal Oak; 3601 13 Mile Rd, Royal Oak, MI, 48073, USA; 2 Department of Pathology, Beaumont Hospital, Royal Oak, MI, USA; 3 Department of Information Technology, Beaumont Health, Southfield, MI, USA; 4 Research Institute, Beaumont Hospital, Royal Oak, MI, USA

**Keywords:** COVID-19, SARS-CoV-2, vaccination, coronavirus, variants, mortality, mechanical ventilation, intensive care unit

## Abstract

**Background:**

While recent literature has shown the efficacy of the SARS-CoV-2 vaccine in preventing infection, it's impact on need for emergency care/hospitalization in breakthrough infections remain unclear, particularly in regions with a high rate of variant viral strains. We aimed to determine if vaccination reduces hospital visits in breakthrough COVID-19.

**Methods:**

This observational cohort analysis compared unvaccinated (UV), partially vaccinated (PV), and fully vaccinated (FV) adult patients with SARS-CoV-2 infection requiring emergency care(EC)/hospitalization within an eight-hospital system in Michigan. Demographic and clinical variables were obtained from the electronic record. Vaccination data was obtained from the Michigan Care Improvement Registry and Centers for Disease Control vaccine tracker. Primary endpoint was rate of emergency care/hospitalization encounters among patients diagnosed with COVID-19. Secondary outcome was severe disease-composite outcome (ICU, mechanical ventilation, or in-hospital death).

**Findings:**

Between December 15,2020 and April 30,2021, 11,834 EC encounters were included:10,880 (91.9%) UV, 825 (7%) PV, 129 (1.1%) FV. Average age was 53.0 ± 18.2 and 52.8% were female. Accounting for the SARS-CoV-2 vaccination population groups in Michigan, the ED encounters/hospitalizations rate relevant to COVID-19 was 96% lower in FV versus UV (multiplicative effect:0.04, 95% CI 0.03 to 0.06, p < 0.001) in negative binomial regression. COVID-19 EC visits rate peaked at 22.61, 12.88, and 1.29 visits per 100000 for the UV, PV, and FV groups, respectively. In the propensity-score matching weights analysis, FV had a lower risk of composite disease compared to UV but statistically insignificant (HR 0.84, 95% CI 0.52 to 1.38).

**Interpretation:**

The need for emergency care/hospitalization due to breakthrough COVID-19 is an exceedingly rare event in fully vaccinated patients. As vaccination has increased regionally, EC visits amongst fully vaccinated individuals have remained low and occur much less frequently than unvaccinated individuals. If hospital-based treatment is required, elderly patients with significant comorbidities are at high-risk for severe outcomes regardless of vaccination status.

**Research in context**

**Evidence before this study**

We searched PubMed on April 5, 2021 for evidence surrounding breakthrough COVID-19 in immunized patients leading to hospital or emergency care encounters using the search terms (SARS-CoV-2 OR novel coronavirus OR COVID-19) AND (vaccination OR immunization) AND (hospitalization OR emergency visit) with no language or time restrictions. Vaccine efficacy has been established by pharmaceutical trials and has triggered the availability of vaccines across the globe. However, effectiveness of vaccines at preventing hospital based treatment for COVID-19 in fully immunized patients in a real world population is poorly understood with no prior publications. Further, it is unclear if vaccines protect patients from severe outcomes associated with COVID-19.

**Added value of this study**

This study is one of the first large, real world investigations addressing the likelihood of fully vaccinated patients requiring hospital-based care in breakthrough COVID-19. We found that ED encounters/hospitalizations related to COVID-19 were 96% lower in fully vaccinated patients compared to unvaccinated patients (multiplicative effect:0.04, 95% CI 0.03 to 0.06, p < 0.001). However, if hospital based care is required for fully vaccinated patients, the risk of severe outcomes is similar to unvaccinated patients.

**Implications of all available evidence**

The need for emergency care/hospitalization due to breakthrough COVID-19 is an exceedingly rare event in fully vaccinated patients. As vaccination has increased regionally, EC visits amongst fully vaccinated individuals have remained low and occur much less frequently than unvaccinated individuals. If hospital-based treatment is required, elderly patients with significant comorbidities are at high-risk for severe outcomes regardless of vaccination status.

Alt-text: Unlabelled box

## 1 Introduction

The COVID-19 pandemic has continued to cause significant morbidity and mortality around the globe with over 146 million cases and 3 million deaths as of April 25, 2021. [1] In December of 2020, the FDA authorized emergency use of the Pfizer-BioNtech vaccine. It became the first of several vaccines to kick off the mass vaccination efforts across the United States. [2] Subsequently, Moderna as well as Johnson and Johnson received emergency use authorization for their vaccines. [3] While preliminary data from safety and efficacy trials have shown positive results, real-world data on its effectiveness is still lacking. [4] Several small cohort studies and one large trial from Israel are currently our only insights into the actual rates of infection, hospitalization, and severe illness among vaccinated individuals. [5], [6], [7] Additionally, as SARS-CoV-2 variants emerge, we are in dire need of more data regarding the effectiveness of our current mass vaccination efforts. [8]

In-vitro studies have shown several variants and mutations to be more transmissible and less sensitive to natural or vaccine-induced antibodies compared to the wild-SARS CoV-2 virus. [8], [9], [10] The Centers for Disease Control (CDC) has published a list of variants of concern and several of them include mutations in the spike protein incorporating the E484K and the L452R substitution. [9,^11^,12] This is highly concerning, particularly in some regions in which new variant cases now outnumber the original wild SARS-CoV-2 strain.

In the latest surge of COVID-19, the state of Michigan has been more severely impacted than the rest of the United States. [1] In Michigan the volume peaked to over 7,000 new daily cases between April 5^th^ and April 12^th^ 2021. [13] According to the CDC, over a 2 week period ending April 24, 2021, 10 SARS-CoV-2 variants were detected within the region. [14] The most common, B.1.1.7 variant, has been identified as the cause of over 50% of new COVID-19 diagnoses in the State of Michigan. [14] While the B.1.1.7 variant has shown to be associated with increased transmissibility, to date there has been no evidence to suggest or negate the impact on vaccine efficacy. However, *in vitro* studies have noted a loss in neutralizing activity by vaccine-induced antibodies when the E484K mutation was introduced to the B.1.1.7 variant. [15]

Vaccination efforts in the State of Michigan have been ongoing since December. [13,16] Given that approximately 42.72% of the state's population was either partially or fully vaccinated as of April 30, 2021, it is unclear if immunization efforts have helped the situation in this recent COVID-19 surge in a population with a high incidence of variant strain disease. [13,^14^,16] Therefore, we aim to evaluate if SARS-CoV-2 vaccination reduces rates of emergency care encounters and hospitalizations. Further we aim to understand vaccination impact on severe illness when breakthrough COVID-19 occurs.

## 2 Methods

### 2.1 Study Design, Setting and Participants

This was a multicenter observational cohort study through electronic health record (EHR; Epic Systems, Verona, WI, USA) analysis to assess vaccine effectiveness on need for emergency care/hospitalizations and severe outcomes in patients with breakthrough COVID-19 comparing fully vaccinated (FV), partially vaccinated (PV), and unvaccinated (UV) patients.

The study was conducted at Beaumont Health, an eight-hospital acute care regional health system caring for 2.2 million people across the communities within the Metro Detroit area. The hospitals range from a large tertiary care academic center to intermediate-sized and smaller community hospitals.

Consecutive adult patients greater than 18 years of age presenting to the emergency department with confirmed COVID-19 as a primary diagnosis were eligible for inclusion. Patients with prior laboratory confirmed SARS-CoV-2 infection, pediatric patients, or those still hospitalized after the designated follow-up date of May 15, 2021 were excluded. The study was approved by the Institutional Review Board at Beaumont Health and registered on clinicaltrials.gov (Identifier: NCT04912700). Written informed consent requirement was waived due to the retrospective nature of this study. Data were analyzed and interpreted by the authors.

### 2.2 Study Definitions

Patients were categorized as either UV, PV, or FV. UV individuals were defined as having positive laboratory SARS-CoV-2 testing with no record of immunization against SARS-CoV-2 or first-dose vaccination after symptom onset. PV individuals were defined as having positive laboratory SARS-CoV-2 testing and symptom onset after a single dose of either mRNA (Pfizer, Moderna) vaccine, or < 14 days after the second dose of either mRNA vaccine (Pfizer, Moderna) or < 14 days after the administration of the single dose of viral vector vaccine (Johnson & Johnson). FV individuals were defined as having positive laboratory testing for SARS-CoV-2 and symptom onset >14 days since administered of second dose of either mRNA vaccine, or >14 days since administration of viral vector vaccine (Johnson & Johnson).

SARS-CoV-2 infection was defined as primary diagnosis of COVID-19 by ICD-10-CM codes in the EHR and either laboratory confirmed positive result (rapid antigen testing or reverse-transcriptase-polymerase-chain-reaction (RT-PCR) by nasopharyngeal swab) or reference to confirmed laboratory diagnosis in the emergency encounter provider note.

### 2.3 Data sources/measurement

EHR data was used to confirm SARS-CoV-2 infection and categorize vaccinated patients. For patients with primary diagnosis of COVID-19 without laboratory confirmed SARS-CoV-2 within the institutional EHR, emergency care provider records were manually reviewed by two independent physicians to confirm outside laboratory diagnosis of infection. Physicians also reviewed provider notes for all PV and FV patients to determine onset of symptoms. The date of symptom onset was used to accurately categorize UV, PV, and FV groups.

Demographic, clinical, and outcomes data were obtained from the EHR. Demographics included age, race, and gender. Clinical data included comorbidities, body mass index (BMI), and number of previous ED visits within the past 6 months. ICD-10-CM codes for comorbidities were used to calculate the Elixhauser comorbidity weighted scores as described by the Agency of Healthcare Research and Quality (AHRQ). [17] Hospital clinical data included level of care required, extracorporeal membrane oxygenation (ECMO), renal replacement therapy, type of oxygen or ventilation therapy, and need for vasopressors.

Hospital admission was based on the clinical judgment of the treating emergency medicine provider and length of stay was calculated for all admitted patients. Discharge disposition post-hospitalization was based on patients’ clinical condition. Patients were either discharged to home, skilled nursing home, rehabilitation facility, hospice, or expired in the hospital.

Vaccination data was made available by the state of Michigan via the Michigan Care Improvement Registry (MCIR) and therefore captured patients who had been vaccinated outside of the Beaumont Health system. [13] This data included vaccine type as well as date of administration. Vaccination prevalence across the population of Michigan was captured weekly via the Centers of Disease Control state-specific vaccine tracker. [14]

### 2.4 Outcome Measures

The primary outcome was rate of emergency care/hospitalization encounters with a diagnosis of COVID-19 among UV, PV, and FV groups. Rate of encounters was defined as number of newly presenting hospital-based COVID-19 within the health system divided by the state population within each respective vaccination group expressed as a rate per 100000 visits. Weekly rates of COVID-19 ED encounters were presented for each vaccination group.

Secondary outcomes included severe disease represented as a composite outcome (ICU admission, mechanical ventilation, or in-hospital death), hospital length of stay, renal replacement therapy, ECMO supplemental oxygen (none, low flow therapy, and high flow therapy), and noninvasive ventilation.

### 2.5 Statistical Analysis

Bivariate analyses were stratified by vaccinated status (UV, PV, FV) using means ± standard deviations and medians with interquartile ranges (IQRs) for continuous variables and frequencies with percentages for categorical variables. Kruskal-Wallis (exact) test (continuous variables) and Chi-squared or Fisher's exact test (categorical variables) were used to compare differences among three categories of vaccination status.

To investigate the effect of vaccination status on the occurrence of COVID-19 emergency care/hospitalization encounters, a negative binomial regression analysis with the log-link, accounting for any potential overdispersion, was used based on weekly rates to the State SARS-CoV-2 vaccination population groups. To characterize variation of the trend in weekly rates across the study period, joinpoint regression analysis (i.e., segmented trend analysis with continuity constraint) through each category of vaccination status on the log-scale with up to 2 joinpoints were used through grid search of joinpoints by Monte Carlo permutation tests. [18]

Cox proportional hazards regression models were used to examine the association between vaccination status and severity of illness, a composite outcome of ICU admission, mechanical ventilation, or in-hospital death. An initial multivariable Cox regression model was built, controlling for demographic characteristics and clinical variables including the Elixhauser weighted score and occurrence of ED visits prior to 6 months. We applied the test for proportionality assumption based on the Schoenfeld residuals. Stratified Cox regression was applied to adjust the potential nonproportional hazards if necessary. In addition, to eliminate the bias of patient characteristics on exposure of vaccination status, we used propensity-score methods to reduce the effect of confounding. The individual propensities for exposure of vaccination status were estimated from a multivariable multinomial logistic regression model that included the same covariates as the multivariable Cox regression. In settings with rare outcomes and unequal exposure distributions of vaccination status, we applied matching weight procedure for assessing the effect of vaccination status on severity of illness. [19] A three-way 1:1:1 nearest neighbor matching was also conducted for evaluating the association. The covariate balance was assessed on matched cohort or the application of matching weight. All tests of statistical significance were indicated with two-sided 95% confidence intervals (CIs) or p < 0.05. Analyses were performed using Joinpoint Regression Program v4.7.0.0, R-4.0.2 (R Foundation for Statistical Computing), and SAS v9.4 (SAS Institute, Inc., Cary, NC).

Ethical approval: This study was approved by the Institutional Review Board at Beaumont Health

Role of the funding source: None

## 3 Results

**Figure 1:**
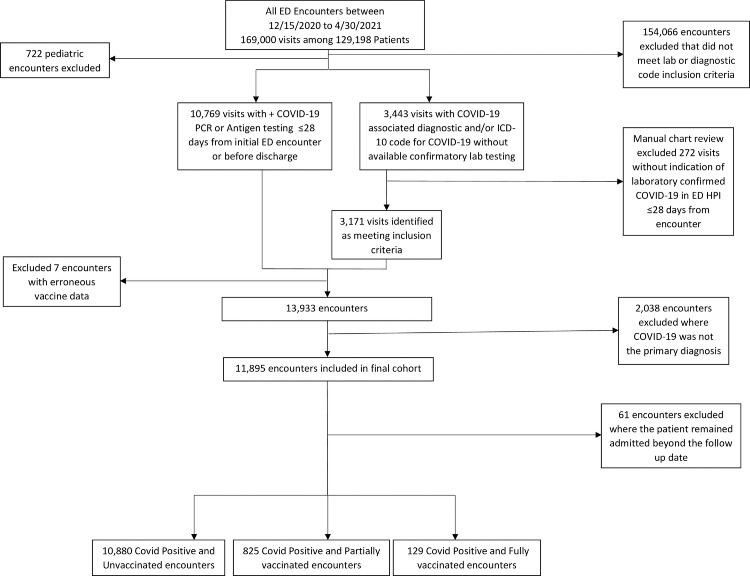
Screening and Categorization of all hospital-based COVID-19 cases into UV, PV, and FV groups Patients with SARS-CoV-2 infection with an ED encounter were eligible participants. Patients with secondary diagnosis of COVID-19 and age less than 18 years were excluded. Patients with primary COVID-19 diagnosis without reference to confirmed testing in the emergency provider note were also excluded. Included patients were then categorized into UV (unvaccinated), PV (partially vaccinated), and FV (fully vaccinated) groups. UV individuals had no record of immunization against SARS-CoV-2 or first-dose vaccination after symptom onset. PV individuals had symptom onset after a single dose of either mRNA (Pfizer, Moderna) vaccine, or < 14 days after the second dose of either mRNA vaccine (Pfizer, Moderna) or < 14 days after the administration of the single dose of viral vector vaccine (Johnson & Johnson). FV individuals had symptom onset >14 days since administered of second dose of either mRNA vaccine, or >14 days since administration of viral vector vaccine (Johnson & Johnson).

Between December 15, 2020 and April 30, 2021 there were a total of 169000 ED encounters within our hospital system. We identified 11895 of these encounters that met our inclusion criteria. After further exclusion of 61 encounters, that remained admitted after our designated follow up date, we were able to analyze 10880 unvaccinated, 825 partially vaccinated, and 129 fully vaccinated ED encounters. (Figure 1).

Complete demographic and comorbidity data for the cohort is displayed in Table 1. There were differences among groups in age, race, ED visits in the prior 6 months, and the Elixhauser comorbidity weighted score. The average ages were 52.1 ± 18.2, 62.5 ± 15.3, and 70.3 ± 16.4 (p < 0.001) for the UV, PV, and FV groups, respectively. There was a larger proportion of African American patients in the UV group at 3452 (31.7%) vs 198 (24%) and 13 (10%) in the PV and FV groups, respectively. The FV group had a statistically higher number of encounters with repeat ED visits within the prior 6 months at 48 (37.2%), vs 196 (23.8%) and 2292 (21.1%) (p < 0.001) in the PV and UV groups, respectively. The average Elixhauser comorbidity weighted scores were 4.3 ± 8.8, 6.7 ± 9.6, and 10.3 ± 11.1 (p < 0.001) in the UV, PV, and FV groups, respectively. Of the 129 FV COVID-19 cases, 20 received JNJ-78436735 (Johnson and Johnson), 39 received mRNA-1273 (Moderna), and 70 received BNT162b2 (Pfizer-BioNtech).

**Table 1:** Patient characteristics by vaccination status for all ED visits

			Vaccination Status						
Variables‡	All		Unvaccinated		Partially Vaccinated		Fully Vaccinated		*p* value
n	11834		10880	(91.9)	825	(7.0)	129	(1.1)	
Age, years	53.0 ± 18.253.0 (39.0, 66.0)		52.1 ± 18.252.0 (38.0, 65.0)		62.5 ± 15.363.0 (52.0, 74.0)		70.3 ± 16.472.0 (62.0, 82.0)		< 0.001
18 to 40-	3053	(25.8)	2983	(27.4)	63	(7.6)	7	(5.4)	
40 to 65-	5542	(46.8)	5126	(47.1)	386	(46.8)	30	(23.3)	< 0.001
≥ 65	3239	(27.4)	2771	(25.5)	376	(45.6)	92	(71.3)	
Sex									
Male	5590	(47.2)	5130	(47.2)	400	(48.5)	60	(46.5)	0.750
Female	6244	(52.8)	5750	(52.8)	425	(51.5)	69	(53.5)	
Race									
White/Caucasian	7134	(60.3)	6467	(59.4)	559	(67.8)	108	(83.7)	
Black/African American	3663	(30.9)	3452	(31.7)	198	(24.0)	13	(10.1)	< 0.001
Other	1037	(8.8)	961	(8.8)	68	(8.2)	8	(6.2)	
BMI, kg/m^2^	32.0 ± 8.631.0 (26.3, 36.2)		32.1 ± 8.731.0 (26.3, 36.2)		32.1 ± 7.931.1 (26.5, 36.8)		30.1 ± 8.428.1 (23.7, 34.1)		0.006
< 30	5340	(45.1)	4898	(45.0)	369	(44.7)	73	(56.6)	0.031
≥ 30	6494	(54.9)	5982	(55.0)	456	(55.3)	56	(43.4)	
Elixhauser weighted score	4.5 ± 8.90.0 (0.0, 10.0)		4.3 ± 8.80.0 (0.0, 9.0)		6.7 ± 9.65.0 (0.0, 13.0)		10.3 ± 11.18.0 (2.0, 16.0)		< 0.001
< 0	2687	(22.7)	2492	(22.9)	178	(21.6)	17	(13.2)	
0 to 10	6538	(55.3)	6099	(56.1)	384	(46.5)	55	(42.6)	< 0.001
> 10	2609	(22.0)	2289	(21.0)	263	(31.9)	57	(44.2)	
ED visits prior to 6 months									
No	9298	(78.6)	8588	(78.9)	629	(76.2)	81	(62.8)	< 0.001
Yes	2536	(21.4)	2292	(21.1)	196	(23.8)	48	(37.2)	

Abbreviations: ED=emergency department; BMI=body mass index.

‡ For continuous variables, means ± standard deviations and medians (interquartile ranges, IQRs) were presented. For categorical variables, frequencies and percentages within parentheses were presented. Missing BMI were less than 5% of observations and imputed by mean.

**Figure 2:**
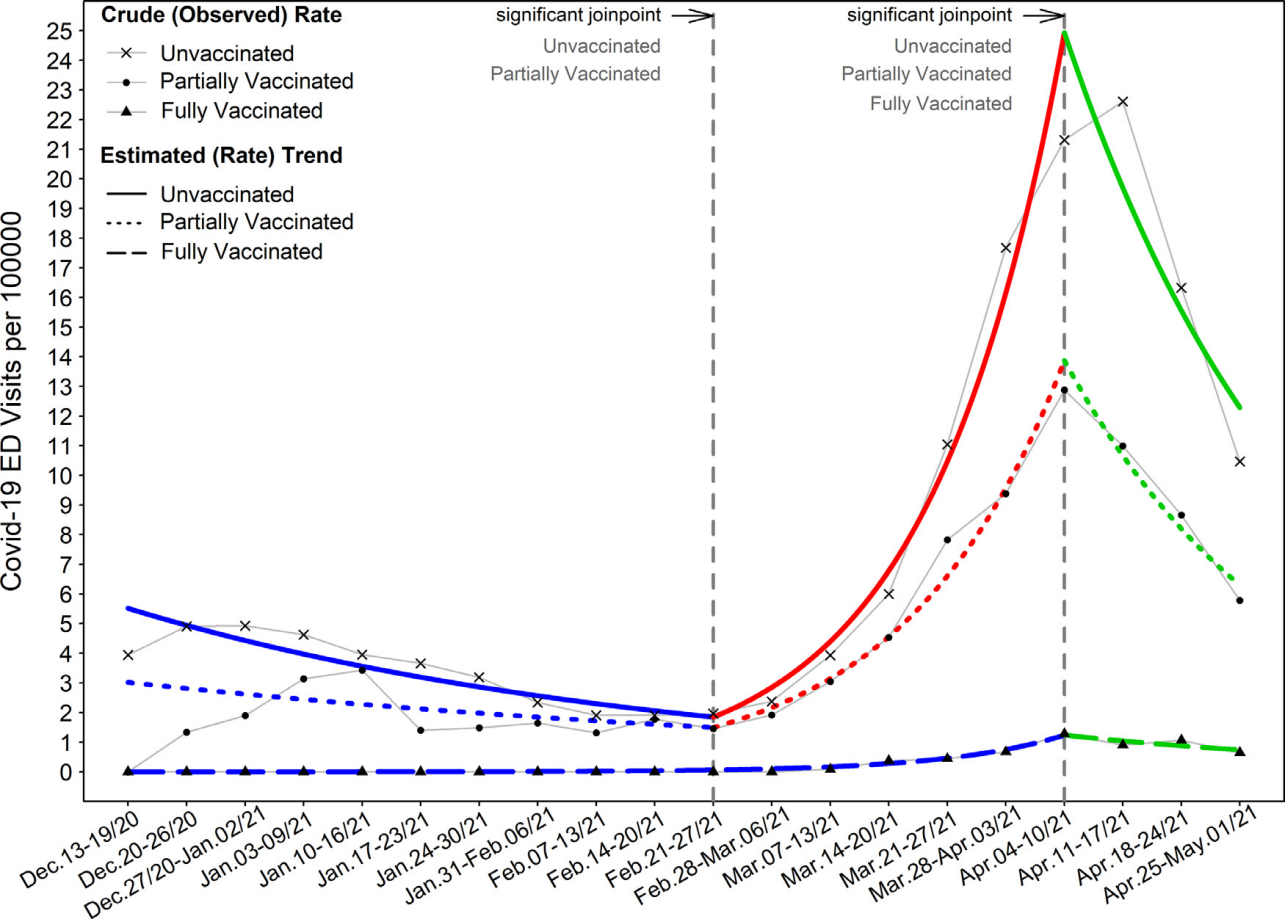
ED encounters of COVID-19 patients among vaccination groups Results shown are for the entire study cohort of adult COVID-19 patients presenting from December 15, 2020 thru April 30, 2021. Case rate of emergency encounters proportionated to the State SARS-CoV-2 vaccination population groups. Weekly crude and estimated trend of COVID-19 infection ED encounters (visits) for each vaccinated group are depicted as number of cases per 100000 over study period. The line graph illustrates the estimated trend of infection ED visits for each vaccinated group. When the state FV (fully vaccinated) population size was only 19 individuals between 12/27/2020 and 1/2/2021, one ED visit occurred in FV group which was not included in analysis due to the bias of an extreme outlier in trend analysis. Through joinpoint regression analysis for each vaccination group, the grid search of joinpoints by Monte Carlo permutation tests identify that statistically significant change of slope estimates occurred before and after specific time points in trend.

We evaluated the effect of vaccination status on the weekly rate of COVID-19 emergency care/hospitalization encounters to the State SARS-CoV-2 vaccination population groups in negative binomial model. Table 2 demonstrates on average, a significant 96% lower rate of COVID-19 ED visits in the FV group compared to UV group (multiplicative effect e^β^: 0.04, 95% CI 0.03 to 0.06, p < 0.001). To characterize variation of the trend across study period, the weekly rate of COVID-19 ED visits to the State SARS-CoV-2 vaccination population groups was depicted for each category of vaccination status (Figure 2). The peak rate of COVID-19 ED visits per 100000 occurred between 4/4/21 and 4/17/21 for all three groups. The crude rate peaked at 22.61, 12.88, and 1.29 visits per 100000 for the UV, PV, and FV groups, respectively. During the increase in SARS-CoV-2 infections presenting to the ED between 2/21 and 4/21, the rate of visits for the fully vaccinated group oscillated between 0.00 to 1.29 per 100000. During this same spike, the rate of visits for unvaccinated individuals went from 1.97 up to 22.61 per 100000 (supplemental Table 1).

**Table 2:** Estimated effect of vaccination status on rate of COVID-19 ED visits from a negative binomial regression

Effects‡^,^§^,^¶			Estimate, β, (SE)	e^β^ (95% CI)	*p* value
Fully vaccinated	vs.	Unvaccinated	–3.20 (0.23)	0.04 (0.03, 0.06)	< 0.001
Fully vaccinated	vs.	Partially vaccinated	–2.55 (0.22)	0.08 (0.05, 0.12)	< 0.001
Partially vaccinated	vs.	Unvaccinated	–0.65 (0.18)	0.52 (0.37, 0.74)	< 0.001

Abbreviations: SE=standard error; CI=confidence interval.

‡ A negative binomial model adjusted for numerical time value (ie, week of COVID-19 ED visits) under the log-link was used to estimate the effect of vaccination status on weekly rates of COVID-19 ED visits in the studied health system, accounting for the weekly state population of each vaccination group (ie, an offset included in regression analysis).

§ When the state FV (fully vaccinated) population size was only 19 individuals between 12/27/2020 and 1/2/2021, one ED visit occurred in fully vaccinated group which was not included in analysis due to the bias of an extreme outlier (supplemental Table 1). Total 59 weekly rates were used.

¶ e^β^ meant the multiplicative effect in the rate.

Patients that experienced severe disease inclusive of composite ICU admission, mechanical ventilation, or in-hospital mortality, had similar baseline characteristics displayed in Table 3. Our composite outcome occurred in 733 (6.8% of 10880) encounters in the UV group, 85 (10.3% of 825) encounters in the PV group, and 16 (12.4% of 129) in the FV group. Among all groups there were 442 (3.7% of 11834) deaths. For each group, death occurred in 384 (3.5% of 10880) UV patients, 50 (6.1% of 825) PV patients, and 8 (6.2% of 129) FV patients.

**Table 3:** Patient characteristics by vaccination status on outcomes of severity of illness for all ED visits

				Vaccination Status						
Severity of illness	Variables‡	All		Unvaccinated		Partially Vaccinated		Fully Vaccinated		*p* value
	n	834		733	(87.9)	85	(10.2)	16	(1.9)	
	Age, years	64.7 ± 15.867.0 (55.0, 75.0)		63.9 ± 16.066.0 (54.0, 75.0)		70.4 ± 11.971.0 (63.0, 79.0)		74.1 ± 16.476.5 (72.0, 84.0)		< 0.001
	Female	366	(43.9)	317	(43.3)	42	(49.4)	7	(43.8)	0.556
Composite§	Race: White/Caucasian	558	(66.9)	493	(67.3)	53	(63.4)	12	(75.0)	
	Black/African American	204	(24.5)	182	(24.8)	21	(24.7)	1	(6.3)	0.111
	Other	72	(8.6)	58	(7.9)	11	(12.9)	3	(18.7)	
	BMI ≥ 30 kg/m^2^	480	(57.6)	427	(58.3)	47	(55.3)	6	(37.5)	0.227
	Elixhauser weighted score	14.8 ± 11.114.5 (6.0, 22.0)		14.7 ± 11.314.0 (6.0, 22.0)		15.9 ± 9.317.0 (8.0, 22.0)		17.2 ± 12.015.5 (11.0, 22.0)		0.388
	ED visits prior to 6 months	209	(25.1)	183	(25.0)	23	(27.1)	3	(18.8)	0.770
	n	708		619	(87.4)	75	(10.6)	14	(2.0)	
	Age, years	63.3 ± 15.465.0 (54.0, 74.0)		62.3 ± 15.564.0 (53.0, 73.0)		69.8 ± 11.870.0 (63.0, 79.0)		74.0 ± 17.679.5 (71.0, 84.0)		< 0.001
	Female	310	(43.8)	266	(43.0)	37	(49.3)	7	(50.0)	0.519
ICU admission	Race: White/Caucasian	475	(67.1)	414	(66.9)	50	(66.7)	11	(78.6)	
	Black/African American	169	(23.9)	151	(24.4)	17	(22.7)	1	(7.1)	0.511
	Other	64	(9.0)	54	(8.7)	8	(10.7)	2	(14.3)	
	BMI ≥ 30 kg/m^2^	419	(59.2)	375	(60.6)	40	(53.3)	4	(28.6)	0.030
	Elixhauser weighted score	14.2 ± 10.914.0 (6.0, 22.0)		13.9 ± 11.114.0 (6.0, 22.0)		16.0 ± 9.517.0 (8.0, 22.0)		16.4 ± 11.815.5 (11.0, 21.0)		0.179
	ED visits prior to 6 months	169	(23.9)	146	(23.6)	20	(26.7)	3	(21.4)	0.820
	n	446		398	(89.2)	42	(9.4)	6	(1.4)	
	Age, years	63.9 ± 14.565.0 (55.0, 74.0)		63.1 ± 14.765.0 (55.0, 73.0)		69.3 ± 11.070.0 (63.0, 78.0)		78.2 ± 6.679.5 (73.0, 84.0)		< 0.001
	Female	202	(45.3)	172	(43.2)	27	(64.3)	3	(50.0)	0.030
Mechanical ventilation	Race: White/Caucasian	282	(63.2)	250	(62.8)	26	(61.9)	6	(100.0)	
	Black/African American	120	(26.9)	107	(26.9)	13	(31.0)	0	(0.0)	0.541
	Other	44	(9.9)	41	(10.3)	3	(7.1)	0	(0.0)	
	BMI ≥ 30 kg/m^2^	299	(67.0)	266	(66.8)	32	(76.2)	1	(16.7)	0.017
	Elixhauser weighted score	15.8 ± 11.116.0 (8.0, 23.0)		15.6 ± 11.116.0 (7.0, 23.0)		17.0 ± 10.617.5 (8.0, 23.0)		19.0 ± 13.414.0 (12.0, 17.0)		0.689
	ED visits prior to 6 months	95	(21.3)	81	(20.4)	11	(26.2)	3	(50.0)	0.123
	n	442		384	(86.9)	50	(11.3)	8	(1.8)	
	Age, years	68.9 ± 13.669.0 (61.0, 79.0)		68.5 ± 13.969.0 (61.0, 78.0)		71.0 ± 11.971.0 (64.0, 82.0)		75.6 ± 5.574.5 (72.0, 79.5)		0.129
	Female	194	(43.9)	163	(42.5)	29	(58.0)	2	(25.0)	0.068
Death	Race: White/Caucasian	292	(66.1)	258	(67.2)	28	(56.0)	6	(75.0)	
	Black/African American	105	(23.8)	88	(22.9)	16	(32.0)	1	(12.5)	0.454
	Other	45	(10.2)	38	(9.9)	6	(12.0)	1	(12.5)	
	BMI (kg/m^2^) ≥ 30	263	(59.5)	226	(58.9)	33	(66.0)	4	(50.0)	0.536
	Elixhauser weighted score	17.6 ± 10.917.0 (10.0, 25.0)		17.8 ± 11.017.0 (10.0, 25.0)		15.7 ± 10.316.5 (8.0, 23.0)		18.5 ± 14.112.5 (11.0, 25.5)		0.499
	ED visits prior to 6 months	108	(24.4)	92	(24.0)	13	(26.0)	3	(37.5)	0.653

Abbreviations: ED=emergency department; ICU= intensive care unit; BMI=body mass index.

§ Composite outcome meant ICU admission, mechanical ventilation, or death.

‡ For continuous variables, means ± standard deviations and medians (interquartile ranges, IQRs) were presented. For categorical variables, frequencies and percentages within parentheses were presented.

We examined the association of vaccination status on severe composite disease (Table 4). The covariate balance achieved a good quality after matching or weighting (supplemental Table 2). In the propensity-score matching weights analysis, results indicate that compared to UV group, FV group had a lower risk of severe composite disease but statistically not significant (hazard ratio HR 0.84, 95% CI 0.52 to1.38); partially vaccinated group was not associated with a significantly higher or lower risk of severe composite disease (HR 1.03, 95% CI 0.78 to 1.35). Propensity-score three-way matching analysis yielded similar conclusion.

**Table 4:** Association between vaccination status and severity of illness

			Crude (Unadjusted) Analysis□		Multivariable Analysis‡^,^□		PS Matching Weights Analysis⁎⁎^,^□		PS Matching Analysis¶^,^□	
			PV vs. UV	FV vs. UV	PV vs. UV	FV vs. UV	PV vs. UV	FV vs. UV	PV vs. UV	FV vs. UV
Severity of illness			HR (95% CI)		HR (95% CI)		HR (95% CI)		HR (95% CI)	
Vaccination Status	Composite§		1.36(1.09, 1.70)−	1.46(0.89, 2.40)NS	1.04(0.83, 1.31)NS	0.89(0.54, 1.46)NS	1.03(0.78, 1.35)NS	0.84(0.52, 1.38)NS	0.92(0.48, 1.77)NS	0.79(0.41, 1.49)NS
	Unmatched	Matched								
UV	773/10880	19/129								
PV	85/825	18/129								
FV	16/129	16/129								
Vaccination Status	ICU admission		1.24(0.98, 1.58)NS	1.28(0.75, 2.17)NS	1.11(0.87, 1.41)NS	1.00(0.59, 1.71)NS	1.13(0.85, 1.51)NS	1.03(0.60, 1.76)NS	1.26(0.56, 2.83)NS	1.27(0.57, 2.81)NS
	Unmatched	Matched								
UV	619/10880	11/129								
PV	75/825	15/129								
FV	14/129	14/129								
Vaccination Status	Mechanical ventilation		1.38(1.00, 1.89)−	1.24(0.55, 2.77)NS	0.92(0.67, 1.27)NS	0.65(0.29, 1.47)NS	0.91(0.62, 1.33)NS	0.71(0.31, 1.63)NS	0.63(0.19, 2.13)NS	0.76(0.24, 2.37)NS
	Unmatched	Matched								
UV	398/10880	8/129								
PV	42/825	5/129								
FV	6/129	6/129								
Vaccination Status	Death		1.36(1.01, 1.82)−	1.42(0.70, 2.86)NS	1.15(0.85, 1.54)NS	1.06(0.52, 2.15)NS	1.05(0.73, 1.52)NS	1.11(0.59, 2.09)NS	0.95(0.44, 2.04)NS	1.07(0.38, 2.99)NS
	Unmatched	Matched								
UV	384/10880	11/129								
PV	50/825	10/129								
FV	8/129	8/129								

Abbreviations: UV=unvaccinated; PV=partially vaccinated; FV=fully vaccinated; ICU=intensive care unit; PS=propensity score; HR=hazard ratio; CI=confidence interval; NS=not statistically significant.

§ Composite severity of illness meant ICU admission, mechanical ventilation, or death.

‡ In multivariable regression analysis, categorical type of patient characteristics listed on Table 1 were covariates. Multivariable Cox regression analysis for the composite severity of illness in all ED patient visits, with stratification on body mass index and Elixhauser weighted score, was adjusted for age, sex, race, and occurrence of ED visits prior to 6 months. For each specific illness (ICU admission, mechanical ventilation, death), multivariable analysis with no stratification was adjusted for age, sex, race, body mass index, Elixhauser weighted score, and occurrence of ED visits prior to 6 months.

⁎⁎ Propensity score matching weights analysis was Cox regression based on the matching weights generalized to the setting of three categories of vaccination status for each individual ED patient visit. Propensity scores in multinomial logistic regression were used to generate matching weights, proposed by Yoshida et al.^19^

¶ Propensity score matching analysis was Cox regression in the three-way matching cohort (n=387).

□ A 95% confidence interval for hazard ratio containing one indicated there was no statistical significance with *p* > 0.05.

Use of ECMO was only seen amongst 4 patients in the UV group with zero instances in the PV and FV groups. The remainder of secondary outcomes by group type are displayed in supplemental Table 3.

## 4 Discussion

Despite aggressive vaccination efforts in Michigan, the rapid increase in new cases during our study period highlights the need to quantify the benefit of these efforts. This study demonstrated that regardless of the high incidence of daily SARS-CoV-2 infections, with a majority due to variant strains, fully vaccinated individuals remained substantially less likely to seek emergency care or become hospitalized. [13,14] Compared to unvaccinated cases, significantly fewer fully vaccinated patients with breakthrough SARS-CoV-2 infection required emergency care and/or hospitalization. Notably, despite the surge of COVID-19 cases during the week of April 4^th^ 2021 the rate of COVID-19 related emergency care for fully vaccinated patients remained low. While this study did not specifically assess for efficacy of the vaccination in preventing disease in the community, this study addressed a possibly more relevant clinical question of likelihood for breakthrough COVID-19 to require hospital-based treatment.

Our cohort of fully vaccinated patients with breakthrough SARS-CoV-2 infections comprised only 1% of COVID-19 emergency care visits during the study period. Within this group, we found that those who required hospitalization and developed severe illness were geriatric patients. Not surprisingly, similar to other vaccinations with reduced effectiveness in the elderly population, this geriatric group represented the population most at risk for serious adverse outcomes. [20,21] Each of the three study groups included patients as young as 19 years of age. In the fully vaccinated group, all 8 deaths and 6 intubations occurred in patients over the age of 65. While in the unvaccinated group, patients as young as 21 died while hospitalized and patients as young as 19 required mechanical ventilation.

When focusing specifically on the composite outcome of severe disease, our data suggests that in the setting of the rare breakthrough infection in a FV patient, vaccination status did not appear to reduce the rate of severe disease. Accompanied with advancing age, chronic disease burden was an important contributing factor to the adverse outcomes in fully vaccinated patients. With an average baseline Elixhauser score >10, this group was at high-risk for near term death after hospital admission regardless of admission diagnosis. Existing literature suggests that a weighted score of 10 predicts a slightly less than 10% risk of in hospital death, while a score of approximately 37 predicts a 50% chance of death while admitted to the hospital. [22] In this fragile group, risk of in-hospital death was similar to that of the matched unvaccinated group, suggesting that vaccination status did not portray an independent reduction in the rate of severe disease. While the mortality rate from COVID-19 has declined from the beginning of the pandemic in which nearly 30% of hospitalized patients died, the death rate was still 6.2% in the fully vaccinated group in our cohort. [23], [24], [25] While this mortality rate is concerning, it is important to understand this outcome in the context of pre-existing risk of in-hospital mortality risk, as determined by the Elixhauser score, as well as of other similar disease processes. For example, comparatively, other endemic respiratory viral illnesses such as influenza which can cause severe disease requiring ICU admission in up to 10% of cases that require hospitalization and mortality rates as high as 8.3%. [26],[27] Despite our hypothesis that vaccination status would yield a reduction in rates of severe disease, it is not surprising that among the elderly population with a high baseline risk of inpatient death of approximately 10%, vaccination status did not provide an independent reduction in severe outcomes. Unfortunately, given the timing of our study, the majority of FV patients requiring hospitalization for breakthrough COVID-19 fell into this category. It is possible that among younger patients with a lower baseline risk of in hospital mortality vaccination status would show an independent benefit for preventing severe disease. It is also important to note that our study only observed patients who developed breakthrough infection that required hospitalization, therefore we cannot comment directly on the efficacy of the vaccines in preventing SARS-CoV-2 infection. However, as we discussed in regard to our primary outcome, despite the surge in COVID-19 cases requiring hospitalization during our study period, the rate of FV patients presenting for emergency treatment remained low. This finding suggests a potential vaccine efficacy for preventing moderate to severe disease within our community.

It is unclear if the vaccination results will hold steady with ongoing viral mutations and emergence of viral variants. Some data suggests that viral mutations may reduce the efficacy of vaccination. For instance, Collier et al. observed a loss in neutralizing activity by vaccine-induced antibodies when the E484K mutation was introduced to the B.1.1.7 variant. This may lead to the need of a substantially larger amount of antibodies to prevent infections.[15] It is also unknown if protective effect of immunization regarding severe disease will wane and expose vulnerable groups to more severe disease. However, our study demonstrated that for now, with over 50% prevalence of variant disease in the region, vaccination is likely effective against existing variants as the rate of breakthrough infections requiring hospital treatment in fully vaccinated patients was low.

Our study had some limitations. The observational cohort study design was a limitation and it is possible that some patients with COVID-19 were not included despite our careful screening of all diagnostic test types. Further, patients with potential COVID-19 with negative laboratory testing were not included in this analysis. As high-risk patients often receive multiple tests to rule out infection, the miss rate was likely small. Additionally, we were unable to determine the rate of prior SARS-CoV-2 infection amongst patients who did not present to our hospitals for emergency care. Therefore, it is possible that in some cases, prior infection, rather than vaccination status, is what reduced the need for emergency care. However, we would assume that prior to vaccine administration, the rates of community acquired COVID-19 was likely similar among all groups and therefore unlikely to alter the rates of emergency care in one group over another. Another limitation was reliance of electronic health record data. The data is reliant on accurate documentation and it is likely that some input errors occurred. Further, some patients had incomplete data and this limited our analysis. Selection bias was another limitation as patients that were still hospitalized after the cutoff follow-up date (May 15) were excluded from the analysis. Fortunately, only 61 (0.5%) patients were still hospitalized. While some of the potentially more severe cases with longer hospital durations were excluded from the analysis, only three cases were excluded due to this reason in the fully vaccinated group, the main population of interest. As the information was time sensitive, we decided it was appropriate to move forward with analysis before waiting for all patient encounters to be complete. Additionally, we assumed vaccination rate of study patients was similar to the published data regarding vaccination status from the state population. It is possible that there were slight variations that were not captured with this methodology. We also assumed our study population had similar rates of variant disease as reported by the state. Test samples were periodically sent to and audited by the state laboratory and internal hospital quality data confirmed our assumptions on rates of variants. Finally, while vaccination data from the state registry was generally robust, in seven cases the data from the MCIR was insensible with some patients receiving a combination of vaccination types or more than two doses. These cases were excluded from the analysis. Additionally, we could not make any vaccination specific conclusions. As the number of fully vaccinated patients was small in our cohort, further sub-analysis by vaccination type was not possible.

In summary, emergency visits and hospitalization in fully vaccinated patients with breakthrough COVID-19 are extremely rare events even in a region with high incidence of variants. When hospitalization occurs, immunized patients are older with many comorbidities. In this high-risk population, risk for severe disease was similar in unvaccinated and vaccinated patients. Future studies are needed to reassess vaccination effectiveness broadly and by type of vaccine as mutations and variants evolve.

## Contributors

AB and SJ designed the study, had full access to all data in the study, and take responsibility for the integrity and accuracy of the data analysis. AB, SJ, MHG, SN, GM, LQ and NWC contributed to study subject enrollment, data collection, data analysis. NWC conducted the formal statistical analysis. All authors contributed to the writing and editing of the manuscript. All authors contributed to data acquisition, data analysis, or data interpretation, and all reviewed and approved the final version of the manuscript. The corresponding author attests that all listed authors meet authorship criteria and that no others meeting the criteria have been omitted.

## Funding

None.

## Declaration of interests

None.

## Acknowledgements

None.

## Data sharing statement

The data that support the findings of this study are available via a data access agreement. Please contact the corresponding author (AB) for this request.

## Appendix Supplementary materials

Image, application 1

Image, application 1

Image, application 1

Image, application 1
