# Supplementary material for: Vaccination reduces need for emergency care in breakthrough COVID-19 infections: A multicenter cohort study
Source: Lancet Reg Health Am. 2021 Sep 9;4:100065. doi: 10.1016/j.lana.2021.100065 (PMC8428472; doi:10.1016/j.lana.2021.100065)
Supplement: Supplementary file 1 [file mmc1.docx]

| Supplementary Table 1. ED encounters (visits) of COVID-19 patients among vaccination status across study period | | | | |
| --- | --- | --- | --- | --- |
|  |  |  | COVID-19  (Beaumont Health System) | |
| Week | Vaccination Status | State Population^‡^ | ED Visits | ED Visits per 100000 |
| 12/13/2020-12/19/2020 | Unvaccinated | 9937009 | 392 | 3.94 |
| 12/13/2020-12/19/2020 | Partially Vaccinated | 21650 | 0 | 0 |
| 12/13/2020-12/19/2020 | Fully Vaccinated | 9 | 0 | 0 |
| 12/20/2020-12/26/2020 | Unvaccinated | 9884058 | 485 | 4.91 |
| 12/20/2020-12/26/2020 | Partially Vaccinated | 74601 | 1 | 1.34 |
| 12/20/2020-12/26/2020 | Fully Vaccinated | 9 | 0 | 0 |
| 12/27/2020-1/2/2021 | Unvaccinated | 9801211 | 483 | 4.93 |
| 12/27/2020-1/2/2021 | Partially Vaccinated | 157438 | 3 | 1.91 |
| 12/27/2020-1/2/2021^¶^ | Fully Vaccinated | 19 | 1 | --^¶^ |
| 1/3/2021-1/9/2021 | Unvaccinated | 9682177 | 448 | 4.63 |
| 1/3/2021-1/9/2021 | Partially Vaccinated | 254546 | 8 | 3.14 |
| 1/3/2021-1/9/2021 | Fully Vaccinated | 21945 | 0 | 0 |
| 1/10/2021-1/16/2021 | Unvaccinated | 9505525 | 376 | 3.96 |
| 1/10/2021-1/16/2021 | Partially Vaccinated | 379248 | 13 | 3.43 |
| 1/10/2021-1/16/2021 | Fully Vaccinated | 73895 | 0 | 0 |
| 1/17/2021-1/23/2021 | Unvaccinated | 9341761 | 342 | 3.66 |
| 1/17/2021-1/23/2021 | Partially Vaccinated | 499064 | 7 | 1.4 |
| 1/17/2021-1/23/2021 | Fully Vaccinated | 117843 | 0 | 0 |
| 1/24/2021-1/30/2021 | Unvaccinated | 9155452 | 292 | 3.19 |
| 1/24/2021-1/30/2021 | Partially Vaccinated | 603419 | 9 | 1.49 |
| 1/24/2021-1/30/2021 | Fully Vaccinated | 199797 | 0 | 0 |
| 1/31/2021-2/6/2021 | Unvaccinated | 9007511 | 211 | 2.34 |
| 1/31/2021-2/6/2021 | Partially Vaccinated | 605256 | 10 | 1.65 |
| 1/31/2021-2/6/2021 | Fully Vaccinated | 345901 | 0 | 0 |
| 2/7/2021-2/13/2021 | Unvaccinated | 8847136 | 169 | 1.91 |
| 2/7/2021-2/13/2021 | Partially Vaccinated | 605183 | 8 | 1.32 |
| 2/7/2021-2/13/2021 | Fully Vaccinated | 506349 | 0 | 0 |
| 2/14/2021-2/20/2021 | Unvaccinated | 8728945 | 167 | 1.91 |
| 2/14/2021-2/20/2021 | Partially Vaccinated | 563161 | 10 | 1.78 |
| 2/14/2021-2/20/2021 | Fully Vaccinated | 666562 | 0 | 0 |
| 2/21/2021-2/27/2021 | Unvaccinated | 8539124 | 168 | 1.97 |
| 2/21/2021-2/27/2021 | Partially Vaccinated | 615842 | 9 | 1.46 |
| 2/21/2021-2/27/2021 | Fully Vaccinated | 803702 | 0 | 0 |
| 2/28/2021-3/6/2021 | Unvaccinated | 8287127 | 197 | 2.38 |
| 2/28/2021-3/6/2021 | Partially Vaccinated | 730819 | 14 | 1.92 |
| 2/28/2021-3/6/2021 | Fully Vaccinated | 940722 | 0 | 0 |
| 3/7/2021-3/13/2021 | Unvaccinated | 7957070 | 313 | 3.93 |
| 3/7/2021-3/13/2021 | Partially Vaccinated | 885674 | 27 | 3.05 |
| 3/7/2021-3/13/2021 | Fully Vaccinated | 1115924 | 1 | 0.09 |
| 3/14/2021-3/20/2021 | Unvaccinated | 7619394 | 457 | 6 |
| 3/14/2021-3/20/2021 | Partially Vaccinated | 1014269 | 46 | 4.54 |
| 3/14/2021-3/20/2021 | Fully Vaccinated | 1325005 | 5 | 0.38 |
| 3/21/2021-3/27/2021 | Unvaccinated | 7285684 | 805 | 11.05 |
| 3/21/2021-3/27/2021 | Partially Vaccinated | 1111003 | 87 | 7.83 |
| 3/21/2021-3/27/2021 | Fully Vaccinated | 1561981 | 7 | 0.45 |
| 3/28/2021-4/3/2021 | Unvaccinated | 6980491 | 1234 | 17.68 |
| 3/28/2021-4/3/2021 | Partially Vaccinated | 1193187 | 112 | 9.39 |
| 3/28/2021-4/3/2021 | Fully Vaccinated | 1784990 | 12 | 0.67 |
| 4/4/2021-4/10/2021 | Unvaccinated | 6540631 | 1394 | 21.31 |
| 4/4/2021-4/10/2021 | Partially Vaccinated | 1242442 | 160 | 12.88 |
| 4/4/2021-4/10/2021 | Fully Vaccinated | 2175595 | 28 | 1.29 |
| 4/11/2021-4/17/2021 | Unvaccinated | 6155909 | 1392 | 22.61 |
| 4/11/2021-4/17/2021 | Partially Vaccinated | 1263720 | 139 | 11 |
| 4/11/2021-4/17/2021 | Fully Vaccinated | 2539039 | 23 | 0.91 |
| 4/18/2021-4/24/2021 | Unvaccinated | 5884715 | 961 | 16.33 |
| 4/18/2021-4/24/2021 | Partially Vaccinated | 1178263 | 102 | 8.66 |
| 4/18/2021-4/24/2021 | Fully Vaccinated | 2895690 | 31 | 1.07 |
| 4/25/2021-5/1/2021 | Unvaccinated | 5672887 | 594 | 10.47 |
| 4/25/2021-5/1/2021 | Partially Vaccinated | 1036800 | 60 | 5.79 |
| 4/25/2021-5/1/2021 | Fully Vaccinated | 3248981 | 21 | 0.65 |
| Abbreviations: ED=emergency department.  ^‡^ Data were extracted up to the update on May 24^th^, 2021.^14^  ^¶^ When the state FV (fully vaccinated) population size was only 19 individuals between 12/27/2020 and 1/2/2021, one ED visit occurred in fully vaccinated group which was not included in analysis due to the bias of an extreme outlier. | | | | |
