# Supplementary material for: Vaccination reduces need for emergency care in breakthrough COVID-19 infections: A multicenter cohort study
Source: Lancet Reg Health Am. 2021 Sep 9;4:100065. doi: 10.1016/j.lana.2021.100065 (PMC8428472; doi:10.1016/j.lana.2021.100065)
Supplement: Supplementary file 2 [file mmc2.docx]

| Supplementary Table 2. Assessment of covariate balance before and after matching or weighting | | | | | | | | | | | | | | | |
| --- | --- | --- | --- | --- | --- | --- | --- | --- | --- | --- | --- | --- | --- | --- | --- |
|  |  | Unmatched Cohort  (Unweighted) | | |  |  | Weighted^⁑^ | | |  |  | Matched Cohort | | |  |
|  |  | Vaccination Status | | |  |  | Vaccination Status | | |  |  | Vaccination Status | | |  |
| Variables^‡^ |  | UV | PV | FV | SMD^¶^ |  | UV | PV | FV | SMD^¶^ |  | UV | PV | FV | SMD^¶^ |
| n |  | 10880 | 825 | 129 |  |  | 129.3 | 128.6 | 129.0 |  |  | 129 | 129 | 129 |  |
| Age, years, mean (SD) |  | 52.1  (18.2) | 62.5  (15.3) | 70.3  (16.4) | 0.72 |  | 68.6  (15.7) | 69.1  (15.2) | 70.3  (16.4) | 0.07 |  | 68.6  (15.1) | 69.0  (14.3) | 70.3  (16.4) | 0.07 |
| Age, years |  |  |  |  |  |  |  |  |  |  |  |  |  |  |  |
| 18 to 40- |  | 2983  (27.4) | 63  (7.6) | 7  (5.4) |  |  | 7.0  (5.4) | 7.7  (6.0) | 7.0  (5.4) |  |  | 7.0  (5.4) | 7.0  (5.4) | 7.0  (5.4) |  |
| 40 to 65- |  | 5126  (47.1) | 386  (46.8) | 30  (23.3) | 0.74 |  | 30.0  (23.2) | 29.9  (23.3) | 30.0  (23.3) | 0.02 |  | 30.0  (23.3) | 30.0  (23.3) | 30.0  (23.3) | < 0.001 |
| ≥ 65 |  | 2771  (25.5) | 376  (45.6) | 92  (71.3) |  |  | 92.3  (71.4) | 91.0  (70.7) | 92.0  (71.3) |  |  | 92.0  (71.3) | 92.0  (71.3) | 92.0  (71.3) |  |
| Sex |  |  |  |  |  |  |  |  |  |  |  |  |  |  |  |
| Male |  | 5130  (47.2) | 400  (48.5) | 60  (46.5) | 0.03 |  | 59.7  (46.2) | 62.0  (49.2) | 60.0  (46.5) | 0.03 |  | 60  (46.5) | 60  (46.5) | 60  (46.5) | < 0.001 |
| Female |  | 5750  (52.8) | 425  (51.5) | 69  (53.5) |  |  | 69.6  (53.8) | 66.6  (51.8) | 69.0  (53.5) |  |  | 69  (53.5) | 69  (53.5) | 69  (53.5) |  |
| Race |  |  |  |  |  |  |  |  |  |  |  |  |  |  |  |
| White/Caucasian |  | 6467  (59.4) | 559  (67.8) | 108  (83.7) |  |  | 108.4  (83.8) | 107.2  (83.3) | 108.0  (83.7) |  |  | 108  (83.7) | 108  (83.7) | 108  (83.7) |  |
| Black/African American |  | 3452  (31.7) | 198  (24.0) | 13  (10.1) | 0.39 |  | 13.0  (10.0) | 12.7  (9.9) | 13.0  (10.1) | 0.02 |  | 13  (10.1) | 14  (10.9) | 13  (10.1) | 0.03 |
| Other |  | 961  (8.8) | 68  (8.2) | 8  (6.2) |  |  | 8.0  (6.2) | 8.8  (6.8) | 8.0  (6.2) |  |  | 8  (6.2) | 7  (5.4) | 8  (6.2) |  |
| BMI, kg/m^2^, mean (SD) |  | 32.1  (8.7) | 32.1  (7.9) | 30.1  (8.4) | 0.16 |  | 30.0  (7.8) | 30.3  (7.4) | 30.1  (8.4) | 0.02 |  | 29.5  (7.6) | 30.1  (7.3) | 30.1  (8.4) | 0.06 |
| BMI, kg/m^2^ |  |  |  |  |  |  |  |  |  |  |  |  |  |  |  |
| < 30 |  | 4898  (45.0) | 369  (44.7) | 73  (56.6) | 0.16 |  | 73.0  (56.5) | 73.1  (56.8) | 73.0  (56.6) | 0.004 |  | 73  (56.6) | 74  (57.4) | 73  (56.6) | 0.01 |
| ≥ 30 |  | 5982  (55.0) | 456  (55.3) | 56  (43.4) |  |  | 56.3  (43.5) | 55.5  (43.2) | 56.0  (43.4) |  |  | 56  (43.4) | 55  (42.6) | 56  (43.4) |  |
| Elixhauser weighted score, mean (SD) |  | 4.3  (8.8) | 6.7  (9.6) | 10.3  (11.1) | 0.40 |  | 9.9  (10.8) | 9.5  (9.9) | 10.3  (11.1) | 0.05 |  | 9.8  (11.2) | 9.4  (9.9) | 10.3  (11.1) | 0.05 |
| Elixhauser weighted score |  |  |  |  |  |  |  |  |  |  |  |  |  |  |  |
| < 0 |  | 2492  (22.9) | 178  (21.6) | 17  (13.2) |  |  | 16.9  (13.1) | 17.4  (13.5) | 17.0  (13.2) |  |  | 17  (13.2) | 18  (14.0) | 17  (13.2) |  |
| 0 to 10 |  | 6099  (56.1) | 384  (46.5) | 55  (42.6) | 0.36 |  | 55.0  (42.5) | 55.5  (43.2) | 55.0  (42.6) | 0.02 |  | 55  (42.6) | 53  (41.1) | 55  (42.6) | 0.02 |
| > 10 |  | 2289  (21.0) | 263  (31.9) | 57  (44.2) |  |  | 57.4  (44.4) | 55.7  (43.3) | 57.0  (44.2) |  |  | 57  (44.2) | 58  (45.0) | 57  (44.2) |  |
| ED visits prior to 6 months |  |  |  |  |  |  |  |  |  |  |  |  |  |  |  |
| No |  | 8588  (78.9) | 629  (76.2) | 81  (62.8) | 0.24 |  | 81.2  (62.8) | 81.1  (63.0) | 81.0  (62.8) | 0.003 |  | 81  (62.8) | 80  (62.0) | 81  (62.8) | 0.01 |
| Yes |  | 2292  (21.1) | 196  (23.8) | 48  (37.2) |  |  | 48.1  (37.2) | 47.5  (37.0) | 48.0  (37.2) |  |  | 48  (37.2) | 49  (38.0) | 48  (37.2) |  |
| Abbreviations: ED=emergency department; BMI=body mass index; UV=unvaccinated; PV=partially vaccinated; FV=fully vaccinated; SD=standard deviation; SMD=standardized mean difference.  ^‡^ For continuous variables, means (standard deviations) were presented. For categorical variables, frequencies (percentages) were presented.  ^⁑^ Propensity scores in multinomial logistic regression were used to generate matching weights, proposed by Yoshida et al.^19^  ^¶^ The standardized mean difference of variables was less than 10% (0.1) threshold to indicate the balance among unvaccinated, partially vaccinated, and fully vaccinated. | | | | | | | | | | | | | | | |
