# Supplementary material for: Vaccination reduces need for emergency care in breakthrough COVID-19 infections: A multicenter cohort study
Source: Lancet Reg Health Am. 2021 Sep 9;4:100065. doi: 10.1016/j.lana.2021.100065 (PMC8428472; doi:10.1016/j.lana.2021.100065)
Supplement: Supplementary file 3 [file mmc3.docx]

| Supplementary Table 3. Treatments and clinical outcomes by vaccination status for hospitalized patients | | | | | | | | | |
| --- | --- | --- | --- | --- | --- | --- | --- | --- | --- |
|  |  |  | Vaccination Status | | | | | |  |
| Variables^‡^ | All | | Unvaccinated | | Partially Vaccinated | | Fully Vaccinated | | *p* value |
| n | 5860 |  | 5250 | (89.6) | 515 | (8.8) | 95 | (1.6) |  |
| **Treatments** |  |  |  |  |  |  |  |  |  |
| Any oxygen therapy | 4502 | (76.8) | 4042 | (77.0) | 396 | (77.0) | 64 | (67.4) | 0.088 |
| Nasal cannula | 2832 | (48.3) | 2563 | (48.8) | 231 | (44.8) | 38 | (40.0) | 0.060 |
| High flow oxygen | 733 | (12.5) | 656 | (12.5) | 67 | (13.0) | 10 | (10.5) | 0.795 |
| Non-invasive ventilation | 494 | (8.4) | 428 | (8.2) | 56 | (10.9) | 10 | (10.5) | 0.080 |
| Mechanical ventilation | 443 | (7.6) | 395 | (7.5) | 42 | (8.2) | 6 | (6.3) | 0.786 |
| ECMO | 4 | (0.1) | 4 | (0.1) | 0 | (0.0) | 0 | (0.0) | 1.000 |
| Renal replacement therapy | 144 | (2.5) | 130 | (2.5) | 14 | (2.7) | 0 | (0.0) | 0.280 |
| Vasopressors | 399 | (6.8) | 348 | (6.6) | 45 | (8.7) | 6 | (6.3) | 0.190 |
| **Outcomes** |  |  |  |  |  |  |  |  |  |
| Hospital length of stay, days | 7.2 ± 6.9  5.2 (3.1, 8.5) | | 7.2 ± 7.0  5.2 (3.1, 8.4) | | 7.3 ± 6.3  5.4 (3.4, 9.0) | | 7.0 ± 5.2  6.1 (3.1, 9.4) | | 0.273 |
| Hospital disposition |  |  |  |  |  |  |  |  |  |
| Home | 4539 | (77.5) | 4110 | (78.3) | 368 | (71.5) | 61 | (64.2) |  |
| Rehabilitation facilities | 79 | (1.4) | 72 | (1.4) | 7 | (1.4) | 0 | (0.0) |  |
| Skilled nursing home | 600 | (10.2) | 514 | (9.8) | 66 | (12.8) | 20 | (21.1) | < 0.001 |
| Hospice | 152 | (2.6) | 125 | (2.4) | 21 | (4.1) | 6 | (6.3) |  |
| Transferred | 54 | (0.9) | 50 | (0.9) | 4 | (0.8) | 0 | (0.0) |  |
| Death | 436 | (7.4) | 379 | (7.2) | 49 | (9.5) | 8 | (8.4) |  |
| Abbreviations: ECMO=extracorporeal membrane oxygenation.  ^‡^ For continuous variables, means ± standard deviations and medians (interquartile ranges, IQRs) were presented. For categorical variables, frequencies and percentages within parentheses were presented. | | | | | | | | | |
