# Supplementary material for: Vaccination reduces need for emergency care in breakthrough COVID-19 infections: A multicenter cohort study
Source: Lancet Reg Health Am. 2021 Sep 9;4:100065. doi: 10.1016/j.lana.2021.100065 (PMC8428472; doi:10.1016/j.lana.2021.100065)
Supplement: Supplementary file 4 [file mmc4.docx]

Supplementary Tables - Captions:

Supplementary Table 1. ED encounters (visits) of COVID-19 patients among vaccination status across study period

Supplementary Table 2. Assessment of covariate balance before and after matching or weighting

Supplementary Table 3. Treatments and clinical outcomes by vaccination status for hospitalized patients
